# Increases in Individualistic Words and Phrases in American Books, 1960–2008

**DOI:** 10.1371/journal.pone.0040181

**Published:** 2012-07-10

**Authors:** Jean M. Twenge, W. Keith Campbell, Brittany Gentile

**Affiliations:** 1 Department of Psychology, San Diego State University, San Diego, California, United States of America; 2 Department of Psychology, University of Georgia, Athens, Georgia, United States of America; Bristol University, United Kingdom

## Abstract

Cultural products such as song lyrics, television shows, and books reveal cultural differences, including cultural change over time. Two studies examine changes in the use of individualistic words (Study 1) and phrases (Study 2) in the Google Books Ngram corpus of millions of books in American English. Current samples from the general population generated and rated lists of individualistic words and phrases (e.g., “unique,” “personalize,” “self,” “all about me,” “I am special,” “I’m the best”). Individualistic words and phrases increased in use between 1960 and 2008, even when controlling for changes in communal words and phrases. Language in American books has become increasingly focused on the self and uniqueness in the decades since 1960.

## Introduction

Just as culture differs across regions, culture changes over time. Trends may appear among individuals in the form of generational change, with each cohort influenced most by the sociocultural environment during their childhood and adolescence (e.g., [1], [2]). Numerous studies have documented generational differences in personality traits, values, and attitudes (e.g., [3]–[7]; for a review, see [8]), with especially consistent increases in individualistic traits such as narcissism, surgency, and positive self-views [9]–[12]. However, there is still debate about whether generational changes exist at all, including in individualism (e.g., [13]).

In addition, cultural change is not limited to the psyches of individuals, and few studies have investigated change in cultural products such as TV shows, song lyrics, and books [14]–[17]. Authors have often documented cultural change using media sources as examples (e.g., [8], [18], [19]), but few of these observations have been verified by empirical data. These trends in cultural products are important because culture includes assumptions and patterns shared by members [20]. Lamoreaux and Morling argue that it is important to study cultural products for at least three reasons [15]. First, culture includes the context as well as the person, and cultural products capture culture “outside the head.” Second, cultural products are not subject to the biases of that potentially plague self-report measures such as social desirability and reference group effects. Third, and perhaps most important, cultural products shape individuals’ ideas of cultural norms and “common sense.” Individuals’ behavior is often influenced by their beliefs about what others in their culture believe and do, even if these assumptions are erroneous (e.g., [21]). Cultural products such as song lyrics, TV shows, and books are likely among the most common sources for perceptions of cultural norms.

Until recently, it was extremely labor-intensive to unearth cultural change through cultural products. However, new technologies such as word coding computer programs (for a review, see [22]) have made it possible to analyze language use in cultural products such as song lyrics [23]. Even then, such studies are very limited in the number of products they can analyze; for example, the song lyrics study examined only the 10 most popular songs for each year between 1980 and 2008. Fortunately, more powerful technology has now made it possible to analyze language use over time in very large numbers of books, another cultural product. The Google Books Ngram Viewer allows users to search a corpus of 5 million books for words and phrases up to 5 words long [24]. The corpus is so large that it would take 80 years for someone to read all of the books for the year 2000 alone [24].

Language use in books could reflect cultural change in at least three ways. First, language use reflects the viewpoints of book authors, showing change in the values and attitudes of an influential portion of the population. Second, books may mirror a market-driven assessment of what people want to read, capturing changes in the preferences of the population of Americans who read books. Third, language use in books may be a microcosm of the language use of people living in that time. For example, a fiction writer may aim to capture realistic modern dialogue. Thus language use in books captures cultural change from the individual level (the author) to the group level (trends in market-based appeal and in language use among the population).

In the current study, we examine language use in American books in English in the modern period (1960–2008; 2008 is the latest data available in Google Books). We chose 1960 as a starting point because many authors have noted that the pace of cultural change in the U.S., particularly in individualism, accelerated beginning in the late 1960s through the 1970s [25]–[28]. Individualistic cultural systems emphasize the rights and importance of the individual self, in contrast to communal cultural systems that emphasize the importance of the group (e.g., [29]). Given past research finding increases in individualism in the U.S., we predicted that the use of individualistic words and phrases would increase. We conducted two studies using the extensive Google Books Ngram database.

We then faced the decision of what language to analyze. That is, which individualistic words (Study 1) and phrases (Study 2) should we examine? We could have generated lists of words and phrases ourselves or asked a selected panel of experts. However, the former approach had the potential for bias given awareness of the research hypotheses, and the second approach is problematic because there is no clear “panel of experts” on this issue, and the selection of such experts would thus be open to bias. Thus we relied on a more objective method, asking a general sample of adults from the website Amazon Mechanical Turk (MTurk) to generate and rate individualistic words and phrases. A sample from the general population also has the possible advantage of better reflecting the views of the average member of the culture than would a group of experts or a group of college students.

Although more objective than a researcher-generated list, a list generated by a current sample still has the limitation of being situated in a particular time. Words and phrases popular at the current moment will be more likely to come to participants’ minds than words and phrases used in the past. This might be best conceptualized as a historical “recency effect.” For example, a particular individualistic phrase (e.g., “all about me”) might show an increase as a result of increasing individualism, but also as a result of accessibility if it is used more often now than it was in the past.

To deal with this potential issue, the sample also generated and rated communal words and phrases as a control. We predict that the individualistic language generated and rated by these modern samples will increase. However, we also predict that individualistic words and phrases will increase even when communal words and phrases are allowed to compete to predict year in a regression equation. With the inclusion of communal words and phrases also generated and rated by a current sample, this method provides a more conservative test of whether individualistic language has increased in American books than simply looking at change in individualistic words and phrases.

## Methods

### Study 1: Individualistic Words

Study 1 examines individualistic and communal words. We identified a list of words by asking a current sample of adults from the general population to generate individualistic and communal words and another sample to rate them on individualism and communalism. We then assessed change in those words over time.

#### Participants and procedure

We used a two-step process to create a sampling of individualistic words. One sample generated words characteristic of individualism, and another rated which were most representative of the concepts. We used the same method to generate communal words.

For both phases, we recruited participants through the online service MTurk, in which participants are paid small amounts to complete various tasks. MTurk samples are typically more diverse in age and ethnicity than college samples or even most other Internet samples, and the data generated meet psychometric standards [30].

Participants in the first phase were 53 adults from the United States aged 20 to 82 (*M* = 37.53, SD = 13.03), of which 39 (73.6%) were women. The ethnicity breakdown was as follows: 67.9% Caucasian, 7.5% African American, 7.5% Asian American, 3.8% Hispanic or Latino, 9.4% of mixed racial heritage, 1.9% Native American, and 1.9% other.

In the generation phase, MTurk participants generated words characteristic of individualism and communalism. Participants were given the following instructions: “We are looking for examples of single words often used in American culture, now and in the past, that express either: A) individualism (defined as focusing on the self and the needs of the self) or B) communalism (defined as focusing on groups, the society, and/or social rules).” Participants were then asked to list five individualistic and five communal words. Eliminating duplicates and foreign words left a list of 105 individualistic words and 137 communal words. We took a conservative approach to similar words, eliminating only plurals (for example, keeping “group” but not “groups”) but retaining noun and adjective forms, as they may have slightly different meanings (for example, “tribe” and “tribal”).

A separate sample of 55 MTurk participants rated the individualistic words on a 1 to 7 scale (with 1 =  “not at all Individualistic” and 7 =  “very individualistic”). Fifty-one other participants rated the communal words on a 1 to 7 scale (with 1 =  “not at all communal” and 7 =  “very communal”). Demographic information was not collected on participants in the second phase.

The 20 top-rated individualistic words were: independent, individual, individually, unique, uniqueness, self, independence, oneself, soloist, identity, personalized, solo, solitary, personalize, loner, standout, single, personal, sole, and singularity. The 20 top-rated communal words were: communal, community, commune, unity, communitarian, united, teamwork, team, collective, village, tribe, collectivization, group, collectivism, everyone, family, share, socialism, tribal, and union.

We then examined change over time in use of these words in the Google Books Ngram database, by far the largest database available of digitized books. These books were likely not truly randomly selected [24]; however, we assume these books were not selected in a way dependent on individualistic and communal word use frequency that also varied systematically with year. As described in more detail by Michel and colleagues, Google used 100 sources such as university libraries and publishers to generate a comprehensive catalog of books [24]. The books were digitally scanned and the corpus was winnowed of serial publications, multiple editions, and books with poor print quality, unknown publication dates, or miscoded language (e.g., a book listed in the library catalog as being written in English that was not actually in English). Country of publication (in this case, the United States) was determined by 100 bibliographic sources [24]. If the books are representative of all titles published in the U.S. in 2002 (the most recent statistics available), 87% are nonfiction and 13% are fiction. This percentage has not differed much over time; in 1960, 12% of books published were fiction [31].

The database reports usage frequency by dividing the number of instances of the word in a given year by the total number of words in the corpus in that year, thus correcting for changes in the number of published works and their length. We analyzed the data using two complementary approaches. First, we simply summed usage means together, with the idea that the natural frequency of the words is relevant for assessing cultural change. In these analyses, a word used more frequently has a proportionally larger influence. In a second set of analyses, we *Z*-scored each word before summing so each word carried an equal weight regardless of absolute frequency. We report data from both analysis strategies.

Usage statistics are available through 2008, though results after 2000 should be interpreted with caution as Google Books was instituted in that year, introducing small changes to the selection of books [24].

### Study 2: Individualistic Phrases

Study 2 examines individualistic phrases. We identified a list of phrases by asking a current sample of adults from the general population to generate phrases and another sample to rate them. The Ngram database includes phrases up to 5 words long. We then assessed change in those words over time.

#### Participants and procedure

The same MTurk sample from Study 1 also generated individualistic and communal phrases under the instructions “We are looking for examples of phrases often used in American culture, now and in the past, that express either: A) individualism (defined as focusing on the self and the needs of the self) or B) communalism (defined as focusing on groups, the society, and/or social rules). These phrases sometimes take the form of advice; other times they express goals.” They were then asked to list five individualistic and five communal phrases. The list was pared of duplicates and some phrases were shortened to 5 words or less (the limit of the Ngram database). A few phrases were eliminated because they showed a use of zero in all years. In the second phase, 59 MTurk participants rated 166 individualistic phrases. In a parallel process, 53 different participants rated 111 communal phrases.

The 20 top-rated individualistic phrases were: all about me, captain of my ship, focus on the self, I am special, I am the greatest, I can do it myself, I come first, I get what I want, I have my own style, I love me, I’m the best, looking out for number one, me against the world, me first, my needs, self love, self reliance, self sufficient, and there’s only one you. The 20 top-rated communal phrases were: all in this together, band together, community spirit, common good, communal living, concern for the group, contribute to your community, it takes a village, sense of community, sharing of resources, strength through unity, the group is very important, the needs of all, together we are strong, united we stand, we are one, we can do it together, work as a team, and working for the whole.

We took the same approach to data analysis as in Study 1, examining both the raw sum of the usage frequencies (so more common phrases would carry more weight) and the sum after *Z*-scoring (with each phrase carrying equal weight).

## Results

### Study 1: Individualistic Words

Individualistic words increased in use in American books between 1960 and 2008. The correlation between year and the sum of the 20 individualistic words was *r*(49)  = .87, *p*<.001. The 20 individualistic words combined made up.096% of words in books published in 1960, and.115% of words in books published in 2008. With an SD of.0063, this is an increase of *d* = 3.02. We also analyzed the data by *Z*-scoring each word before summing them. The correlation between publication year and the sum of the Z-scores was *r*(49)  = .86, *p*<.001 for the 20 individualistic words, very similar to the simple sum.

Words with especially linear increases (*r* >.80) included “identity,” “personalized,” “personalize,” “self,” “standout,” and “unique.” Other words decreased in use, including “independent,” “independence,” “individual,” “sole,” and “solitary.”

When both individualistic and communal words are included in a regression equation predicting year, only individualistic words are significant (Beta  = .83, *p*<.001; for communal words, Beta  = .05, *ns*). When the *Z*-scored sums of both the individualistic and communal words were included in a regression equation predicting year, individualistic words increased while communal words decreased (Beta  = .84, *p*<.001; for communal words, Beta  =  −.15, *p*<.05). Thus when the common variance of being generated by a modern sample is partialled out, only individualistic words have increased since 1960.

The bivariate correlation between publication year and the simple sum of the 20 communal words was *r*(49)  = .69, *p*<.001. The 20 communal words combined increased from.121% in 1960 to.133% in 2008 (SD = .0068), an increase of *d* = 1.91. However, communal words did not change significantly when the sum of the *Z*-scores is used instead, *r*(49)  =  −.26, *p* = .07. Thus when each word is weighted equally, communal words were unchanged 1960–2008.

The differences between the analyses of sums and Z-scores are due to the influence of more frequently used words. Many of the more frequently used words (e.g., family, share) increased in use 1960–2008. These words exert a greater influence in the summed analyses (which increased), but not in the Z-score analyses (which showed no change).

All results were similar when restricted to the data before 2000.

### Study 2: Individualistic Phrases

Individualistic phrases increased in use in American books between 1960 and 2008. The correlation between year and the sum of the 20 individualistic phrases was *r*(49)  = .90, *p*<.001. Individualistic phrases increased from.000093% in 1960 to.00016% in 2008 (*SD* = .000022), *d* = 3.05. Analyses using the sum of the *Z*-scores for the phrases also produced a significant positive correlation between year and individualistic phrases, *r*(49)  = .92, *p*<.001.

When both individualistic and communal phrases are included in a regression equation predicting year, only individualistic phrases are significant, though communal phrases showed a marginal trend (Beta  = .71, *p*<.001; for communal phrases, Beta  = .23, *p* = .06). Thus when the common variance of being generated by a current sample is partialled out, only individualistic phrases have significantly increased since 1960. When the *Z*-scored sums of both the individualistic and communal phrases were included in a regression equation predicting year, both individualistic and communal phrases increased, though communal phrases showed a weaker effect (Beta  = .76, *p*<.001; for communal words, Beta  = .22, *p*<.01).

The correlation between year and the sum of the 20 communal words was *r*(49)  = .83, *p*<.001. Communal phrases increased from.00032% in 1960 to.00057% in 2008 (SD  = .000087), *d* = 2.87. The results were similar for the sum of the *Z*-scores, *r*(49)  = .77, *p*<.001.

All results were similar when restricted to the data before 2000.

## Discussion

The use of both individualistic words (Study 1) and phrases (Study 2) increased over time in a very large corpus of books in American English. This increase remained significant even when a sample of communal words and phrases also generated by a modern sample was controlled for statistically.

We interpret these changes in published language as reflecting broader cultural changes. That is, we believe these data provide further evidence that American culture has become increasingly focused on individualistic concerns since 1960. Using cross-temporal data to assess cultural change over time within one country is similar to using cross-cultural data to assess differences between cultures during the same historical period. Thus, America today is culturally distinct from America in 1960– at least in the realm of individualism.

It is also interesting to consider the specific words and phrases generated by the current sample. This list may provide a view of modern language relevant to individualism and communalism. Within the individualistic words, variations on the word “personal” were common. Many of the individualistic phrases, especially those that increased over time, included the word self or emphasized uniqueness and/or being better than others, consistent with the rise in these traits among individuals (e.g., [11], [12], [32]). However, individualistic words and phrases emphasizing standing alone (such as independence, self reliance, self sufficient, solitary, and sole) were among the few that decreased or did not change. The modern communal words and phrases prominently featured the words “community” and “team,” constructions of communalism that were apparently used less often in previous decades. This is a potentially interesting avenue for future research.

The results also showed a stronger trend for communal phrases than for communal words. This may be a function of the communal phrases generated by our current sample; more of the phrases (vs. the words) involved modern concepts such as teamwork and community. Perhaps because they are more complex and distinct, phrases may pass in and out of fashion more quickly than words; thus, they may be more subject to a “recency effect” with more recently popular phrases generated by our current sample. This may have produced the larger increase in communal phrases versus communal words. It is also possible that the current sample may have generated phrases more extreme in individualism but less extreme in communalism.

### Limitations and Future Research

We want to raise several notes of caution regarding these data. First, it is important to point out that (a) the change in individualism was smaller when communal words and phrases were controlled and (b) communal words and phrases also increased – at least when assessed in isolation. Thus, a major part of the increase we found likely reflects the use of a present-day sample to generate and rate terms reflecting both individualism and communalism, with the words and phrases likely reflecting current language use (what we referred to as a cultural “recency effect”). Given this, the true increase in individualism is likely significantly smaller than the simple correlations or *d*’s reflect. Instead, the smaller, semi-partial correlations for individualism controlled for communal words and phrases (.86 and.71, respectively, from the regression equations) or perhaps differences in *d*’s between individualistic and communal words and phrases (about *d* = .50) are better approximations of the change.

We also want to be clear that these results do not rule out increasing communalism in American books. The question, then, is which analysis – individualistic words/phrases in a bivariate equation or controlled for communal words/phrases – is more representative of change, with one more liberal and the other more conservative. We believe the issue of communal language change needs greater study before strong conclusions can be drawn.

In these studies, a current sample of Americans from the general population generated and rated individualistic and communal words and phrases. This had the strength of being objective (as opposed to generating the words and phrases ourselves). However, this was by necessity a modern sample, which then generated modern words and phrases, most of which increased over time. Our solution was to use a sample of communal words and phrases as a control. Although it is not possible to have past samples generate or rate word lists, future research could employ different strategies to generate and rate word lists. For example, samples of people over 60 years old could generate words and phrases, on the theory that they might be more likely to generate words and phrases used in the past. Another possibility is for older people to generate lists of words and phrases they remember being popular in their youth, versus those popular at the moment. Each of these methods presents its own biases. When identifying cultural changes, we suggest multiple studies by multiple groups of researchers, using converging methods with multiple data forms. Over time, this will provide the most thorough picture of change.

We should also note that the sample that generated and rated the words and phrases was American, as was the Ngram American English corpus of books from which we drew. This was a purposeful choice, as we were interested in cultural change within one culture. Nevertheless, this means the results cannot be generalized to other cultures. Future research should explore whether cultural products in other countries and cultures also reflect a rise in individualism. Such research could also examine cross-cultural differences in language use in books. For example, the communal words and phrases were more commonly used than the individualistic words and phrases. This seems paradoxical, as the U.S. is a highly individualistic country; however, the communal value of benevolence is ranked highest around the world, even in the U.S. [33]. Thus it is entirely possible that communal words and phrases are used more frequently around the world – and likely even more frequently in more communal and less individualistic nations. Until future research compares language use in the U.S. with that in another country or countries, however, we cannot conclude anything about the relative individualism or communalism of the U.S. from these data.

### Conclusions

This study demonstrates that language use in books reflects increasing individualism in the U.S. since 1960. Language use in books reflects the larger cultural ethos, and that ethos has been increasingly characterized by a focus on the self and uniqueness.
